# Use of Antibody Tools to Provide Serologic Evidence of Elimination of Lymphatic Filariasis in The Gambia

**DOI:** 10.4269/ajtmh.17-0371

**Published:** 2017-11-20

**Authors:** Kimberly Y. Won, Sana Sambou, Amanda Barry, Keri Robinson, Momodou Jaye, Bakary Sanneh, Abdoulie Sanyang, Katherine Gass, Patrick J. Lammie, Maria Rebollo

**Affiliations:** 1 Centers for Disease Control and Prevention, Atlanta, Georgia;; 2 Swiss Tropical and Public Health Institute, Basel, Switzerland;; 3 University of Basel, Basel, Switzerland;; 4 Ministry of Health and Social Welfare, Banjul, The Gambia;; 5 Task Force for Global Health, Decatur, Georgia

## Abstract

A current need in the global effort to eliminate lymphatic filariasis (LF) is the availability of reliable diagnostic tools that can be used to guide programmatic decisions, especially decisions made in the final stages of the program. This study conducted in The Gambia aimed to assess antifilarial antibody levels among populations living in historically highly LF-endemic areas and to evaluate the use of serologic tools to confirm the interruption of LF transmission. A total of 2,612 dried blood spots (DBSs) collected from individuals aged 1 year and above from 15 villages were tested for antibodies to Wb123 by enzyme-linked immunosorbent assay (ELISA). A subset of DBS (*N* = 599) was also tested for antibodies to Bm14 by ELISA. Overall, the prevalence of Wb123 was low (1.5%, 95% confidence interval [CI] 1.1–2.1%). In 7 of 15 villages (46.7%), there were no Wb123-positive individuals identified. Individuals with positive responses to Wb123 ranged in age from 3 to 100 years. Overall, Bm14 prevalence was also low (1.5%, 95% CI 0.7–2.8%). Bm14 positivity was significantly associated with older age (*P* < 0.001). The low levels of antibody responses to Wb123 observed in our study strongly suggest that sustainable LF transmission has likely ceased in The Gambia. In addition, our results support the conclusion that serologic tools can have a role in guiding programmatic decision making and supporting surveillance.

## INTRODUCTION

Lymphatic filariasis (LF) is a mosquito-transmitted parasitic disease caused by three main species of filarial worms (*Wuchereria bancrofti*, *Brugia malayi*, and *B. timori*).^1^ In 1997, at the 50th World Health Assembly (WHA), a resolution was passed to eliminate LF as a public health problem by 2020 (WHA resolution 50.29).^2^ Shortly thereafter, in 2000, the Global Program to Eliminate Lymphatic Filariasis (GPELF) was organized to assist countries in achieving this goal.^3^ At the onset of GPELF, it was estimated that 120 million individuals were infected and that approximately 1.3 billion people throughout the tropics and subtropics were at risk of filarial infection.^3^ To reach the established elimination targets, LF programs set out to treat individuals in endemic areas through annual community-wide mass drug administration (MDA) for at least 5 years. By the end of 2015, MDA had been implemented in 63 of 73 LF-endemic countries, with a cumulative total of 6.2 billion treatments delivered since the launch of GPELF.^4^

Typically, LF programs first conduct mapping surveys to identify areas to target for treatment, then conduct multiple rounds of MDA, and finally conduct transmission assessment surveys (TAS) designed to determine whether infection levels are low enough to stop MDA.^5^ Although most LF-endemic countries have successfully followed this approach, some countries have yet to scale-up programmatic activities. Although there is a clear need to implement MDA in some of these areas, others have a history of high microfilariae (mf) prevalence; but recent surveys have failed to confirm the presence of infection and appropriate programmatic action is unclear. A current need in the global effort to eliminate LF is the availability of reliable diagnostic tools that can be used to guide programmatic decisions, especially decisions made in the final stages of the program. In addition, validated tools are needed to confirm the absence of LF transmission in situations where the requirement for program implementation is unclear.

In the early stages of the global LF program, detection of mf in the peripheral blood was used routinely to monitor the impact of MDA.^3,6,7^ In most LF-endemic areas in the world where the parasite is nocturnally periodic, logistic challenges were encountered because of the requirement to collect blood at night between 22:00 and 02:00 hours. Furthermore, it became increasingly difficult to detect mf in populations after multiple rounds of MDA.^8^ Many of the limitations experienced with mf detection were addressed with the introduction of the immunochromatographic card test (ICT) to detect circulating filarial antigen (CFA).^9^ Importantly, the ICT could be conducted with blood collected at any time of the day, eliminating the need for night blood collections. Based on the results of a multicountry comparison, the ICT was the diagnostic tool recommended for TAS.^8^ Currently, the recently introduced Filariasis Test Strip (FTS)^10^ is the official diagnostic tool recommended for TAS. As production of the ICT is being phased out, it is acceptable for LF programs to use either the ICT or FTS in the interim.

Although tools to detect CFA have been and continue to be useful for the global LF program, there are some limitations to their use. Similar to the observed decline in mf prevalence after treatment, antigenemia also begins to decline and becomes increasingly difficult to detect in populations that have been subjected to multiple rounds of MDA.^8^ In addition, as infection prevalence declines, the prevalence and magnitude of serologic responses shifts, and operational sensitivity of the assays will decline compared with the laboratory-defined sensitivity. Recent evidence suggests that the detection of antifilarial antibodies provides the earliest indicator of filarial exposure,^11^ and the absence of detectable antibody responses may provide evidence that transmission has been interrupted. As control programs move ahead, there will be fewer infection-specific antibody responses in populations, and increasingly, only residual antibody responses in older individuals will be observed. Many of the currently available LF antibody tests have been shown to be sensitive measures of exposure and infection, but may lack the specificity needed to make important programmatic decisions.^12–14^ However, the identification of a highly specific recombinant antigen, Wb123,^15^ as an early serologic marker for filarial infection provides a new surveillance tool^16^ that may be useful to confirm the interruption of LF transmission.

In the 1950s, mf prevalence in The Gambia was reported to be approximately 50%,^17^ among the highest in the world. Surveys conducted in the 1970s showed that mf prevalence had declined significantly in the absence of any LF-specific interventions.^18^ In 2001, stored serum samples from individuals living in historically highly LF-endemic areas were tested for the presence of CFA, a more sensitive diagnostic marker than mf,^8^ and results indicated an even further decline in LF prevalence compared with results from the 1970s.^19^ In 2003, the Gambian Ministry of Health and Social Welfare (MOHSW) conducted a national LF mapping survey with the intent of identifying areas in need of MDA. Interestingly, the results indicated that MDA was not necessary.^19^ In 2013, the MOHSW conducted TAS and found no evidence of LF transmission among young children.^19^ Although The Gambia has not followed the traditional approach for LF elimination, it seems that the current criteria used as the operational definition of elimination have been achieved. The absence of antigenemia among children was likely an indicator of interrupted transmission, but there was no information collected from older age groups. In the absence of LF-specific interventions, the Gambian MOHSW believed it was important to assess LF status among older age groups to complement the TAS results. The current study aimed to assess antifilarial antibody levels among communities living in historically highly LF-endemic areas of The Gambia and to use serologic tools to determine whether interruption of LF transmission has been achieved.

## METHODS

### Study site and design.

The study took place in February 2015 in The Gambia, a small African country with the Atlantic Ocean to the west and all of its land borders shared with Senegal. It is the smallest country on the mainland of the continent and has an estimated population of 1.9 million people. The country is divided into five divisions (Central River, Lower River, North Bank, Upper River, and Western) and one city (Banjul). Fifteen villages (Dampha Kunda, Jappineh, Jambanjelly, Jiboroh Koto, Kafuta, Kamanka, Kembujeh, Keneba, Kololi, Latrikunda Sabiji, Mandinaring, Marakissa, Sare Opatah Jawa/Darsilameh, Sikon Batabu Kantora, and Tambasansang) with the highest historic evidence of LF were purposely selected for this study. The villages were located in four of the five divisions and in Banjul. No villages in the Central River Division were included in the study because LF prevalence in this area was low in the 1970s, and no evidence of infection was found in recent surveys. Following World Health Organization (WHO) guidance for monitoring LF in sentinel sites,^5^ a convenience sample of approximately 300 individuals (≥ 1 year old) in each village was enrolled in the study.

### Ethical considerations.

The study was approved by the Gambia Government/MRC Joint Ethics Committee. The Institutional Review Board of the U.S. Centers for Disease Control and Prevention (CDC) determined CDC to be a nonengaged research partner. Study details were explained to potential participants and written informed consent was obtained from persons who agreed to participate. Parents or guardians provided permission for participation of children < 18 years. In addition, children aged between 7 and 17 years were asked to provide verbal assent for their participation. All identifiable information was kept confidential and maintained using a secure database with access restricted to essential study personnel.

### Data collection.

On the day of sample collection, residents of the community were asked to come to a central location within the village. On obtaining informed consent, participants were assigned a unique identifier and asked to provide basic demographic information such as age and sex. All data were collected on Android platform smartphones (BLU, Miami, FL) using the LINKS application^20^ and uploaded to a secure SQL server.

### Blood collection and antigen testing.

Approximately 100 µL of blood was collected using a finger stick and used for the detection of CFA by ICT (Alere, Scarborough, ME). The cards were read at 10 minutes and marked as either positive or negative according to the manufacturer’s instructions. An additional 60 µL of blood (10 µL per extension × 6 extensions) was collected onto the filter paper (Cellabs, Sydney, Australia) and dried and stored for antifilarial antibody testing. The dried blood spots (DBSs) were stored at −20°C until shipped to the CDC for testing.

### Antibody testing by enzyme-linked immunosorbent assay (ELISA).

DBSs were tested with the Filaria Detect™ IgG4 ELISA (InBios, Seattle, WA), a direct enzyme immunoassay that detects IgG4 antibodies to the recombinant Wb123 antigen. This test was performed according to the protocol provided by the manufacturer with minor modifications. Briefly, one blood spot extension (10 µL whole blood) was eluted overnight in 250 µL of the sample dilution buffer provided in the kit to yield an approximate 1:50 serum dilution. The following day, the samples were tested in duplicate by adding 100 µL of eluate to each well. Kit-provided positive and negative controls were also tested in duplicate at a 1:50 dilution. In addition, two internal positive controls (H3, H19; not provided in the kit) available at the CDC were tested in duplicate at 1:1,500 (H3) and 1:900 (H19) dilutions, respectively, on each plate. These internal controls were used to standardize results across plates. The plates were incubated at 37°C for 30 minutes and then washed with the kit-provided wash buffer. Mouse anti-human IgG4 conjugated with horseradish peroxidase (HRP) was added to each well at a 1:100 dilution and incubated at 37°C for 30 minutes. After washing, 100 µL of tetramethylbenzidine (TMB) was added to each well, and plates were developed at room temperature in the dark for 9 minutes. Reactions were stopped by adding 50 µL of the kit-provided stop solution to each well, and plates were read at 450 nm. To compare optical density (OD) values between Wb123 ELISA plates, mean OD values for each sample were divided by the mean OD of the H3-positive control to normalize the results.

All available samples from two villages (Kololi and Tambansansang) were tested with a noncommercial ELISA (CDC; Atlanta, GA) for IgG4 antibodies against the recombinant Bm14 antigen.^21^ This assay has relatively high sensitivity (92%) and specificity (99%) and was determined to be an appropriate alternative to a commercially available Bm14 ELISA. Greiner microlon high-binding plates (Greiner Bio-One, Monroe, NC) were coated with Bm14 at a concentration of 0.3 µg/mL in antigen sensitizing buffer (0.5 M Tris/HCl, pH 8.0 + 0.3 M KCl + 2 mM EDTA) and incubated overnight at 4°C. One blood spot extension was added to 250 µL of the dilution buffer (PBS pH 7.2 + 0.3% Tween20 + 5% milk) and incubated overnight at 4°C. Positive control serum samples were diluted 1:50 in the dilution buffer and used to construct an 9-point standard curve and to serve as two calibrator positive controls for each plate. All controls were held at 4°C overnight. The following day, the diluted samples and controls were tested in duplicate by adding an aliquot of 100 µL to each well. Plates were incubated at room temperature on a shaker for 30 minutes. Mouse anti-human IgG4 conjugated with HRP (cloneHP6025; Southern Biotech, Birmingham, AL) was diluted at 1:2,000 and 100 µL was added to each well. Plates were incubated at room temperature on a shaker for 30 minutes. TMB (100 µL) was added to each well and plates were developed at room temperature for 2 minutes. One hundred microliters of 1 M H_2_SO_4_ was added to each well to stop the reaction, and plates were immediately read at 450 nm. Plates were washed between each step with PBS + 0.3% Tween20.

### Cutoff determination for ELISAs.

Cutoff values for Wb123 and Bm14 were calculated at CDC from receiver operator characteristic curves using sera from *W. bancrofti* mf positive patients and presumed negative sera from adult US citizens with no history of foreign travel to LF-endemic countries.

### Statistical analysis.

Statistical analyses were performed in Stata version 14.1 (StataCorp LP; College Station, TX) and used the 5% level of significance. χ^2^ tests and logistic regression were used to identify associations between seropositivity and other factors.

## RESULTS

**Figure 1.**
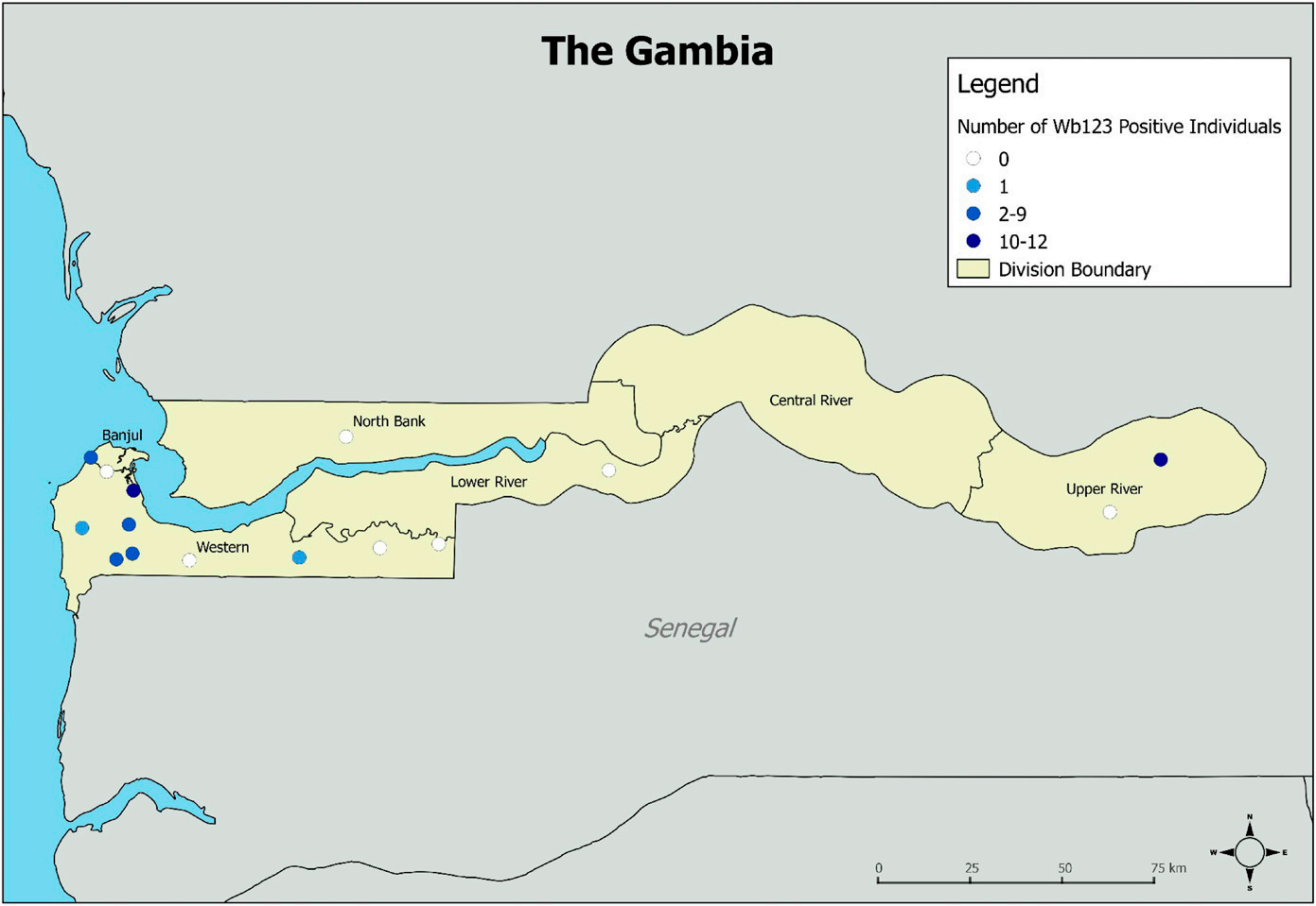
Location of the 15 study villages in The Gambia and Wb123 antibody status in 2015. This figure appears in color at www.ajtmh.org.

A total of 4,481 individuals (aged 1–100 years) from the 15 villages were enrolled in the study. Of those enrolled, a total of 2,612 (58.2%) DBS from all the 15 villages were tested for antibodies to Wb123. There was no difference in age or sex between individuals not included for serologic testing and individuals with antibody results. Demographic information was not available for 161 (6.2%) samples with antibody results. Antibody prevalence for individuals with missing demographic data was not different from prevalence for those with available demographic information. There were no individuals who were antigen positive by ICT. Overall, the prevalence of positive Wb123 responses was low (1.5%, 95% confidence interval [CI] 1.1–2.1%). In 7 of 15 villages (46.7%), there were no antibody-positive individuals identified. Of the eight villages with at least one person with a positive Wb123 result, six (75%) were located in the Western Division (Figure 1). Individuals with positive responses to Wb123 ranged in age from 3 to 100 years. Wb123 results by community are summarized in Table 1. There was no statistically significant difference in Wb123 prevalence among the study villages once adjusted for age, sex, and clustering by village.

**Table 1:** Wb123 antibody prevalence by community in The Gambia in 2015

Division	Community	Total enrolled	Total tested	Median age in years (range)	Wb123 positive	% Positive [95% CI]
Banjul	Kololi	335	307	10 (1–70)	2	0.7 [0.1, 2.3]
Banjul	Latrikunda Sabiji	305	124	17 (1–70)	0	0.0 [0, 2.9*]
Lower River	Jappineh	308	123	10 (1–80)	0	0.0 [0, 3.0*]
Lower River	Keneba	305	124	10 (1–80)	0	0.0 [0, 2.9*]
North Bank	Sare Opatah Jawa/Darsilameh	309	118	11 (1–90)	0	0.0 [0, 3.1]
Upper River	Dampha Kunda	342	124	8 (1–89)	0	0.0 [0, 2.9*]
Upper River	Tambasansang	294	292	12 (1–100)	10	3.4 [1.7, 6.2]
Western	Jambanjelly	235	90	11 (1–70)	1	1.1 [0, 6.0]
Western	Jiboro Koto	231	90	11 (1–70)	3	3.3 [0.7, 9.4]
Western	Kafuta	305	124	9.5 (1–70)	0	0.0 [0, 2.9]
Western	Kamanka	305	123	12 (2–80)	0	0.0 [0, 3.0]
Western	Kembujeh	303	125	10 (1–80)	4	3.2 [0.9, 8.0]
Western	Mandinaring	299	296	10.5 (1–100)	12	4.1 [2.1, 7.0]
Western	Marakissa	302	275	6 (1–90)	4	1.5 [0.4, 3.7]
Western	Sikon Batabu Kantora	303	116	13 (1–90)	1	0.9 [0, 4.7]
#N/A (missing demographic data)	–	–	161	N/A	3	1.9 [0.4, 5.3]
–	Total	4,481	2,612	11 (1–100)	40	1.5 [1.1, 2.1]

* One-sided, 97.5% confidence interval.

All available samples from Kololi and Tambasansang were also tested by Bm14 ELISA. Overall, Bm14 prevalence was low (1.5%, 95% CI 0.7–2.8%) in these two villages. In Kololi, there were two Wb123-positive individuals, but there were no positive Bm14 responses in this community. In Tambasansang, there were 10 (3.4%) Wb123-positive individuals ranging in age from 4 to 65 years. Although a similar number of Bm14 positive persons was identified (9/292; 3.1%), all Bm14-positive individuals were older than 50 years. Bm14 positivity was significantly associated with older age (*P* < 0.001). The results of antibody testing and historic mf results are summarized in Table 2.

**Table 2:** Microfilariae prevalence in selected villages of The Gambia in the 1970s^18^ and antifilarial responses to Wb123 and Bm14 in the same villages in 2015

	Kololi					Tambasansang				
	1974–1976		2015			1974–1976		2015		
Age	*n*	Mf [95% CI]	*n*	Wb123 [95% CI]	Bm14 [95% CI)	*n*	Mf [95% CI]	*n*	Wb123 [95% CI]	Bm14 [95% CI]
0–5	N/A	–	52	0.0 [0, 6.8*]	0.0 [0, 6.8*]	N/A	–	61	3.3 [0.4, 11.3]	0.0 [0, 5.9*]
6–15	44	22.7 [11.5, 37.8]	169	1.2 [1.4, 4.2]	0.0 [0, 2.2]	107	17.8 [11.0, 26.3]	121	2.5 [0.5, 7.1]	0.0 [0, 3.0]
16+	38	26.3 [13.4, 43.1]	86	0.0 [0, 4.2*]	0.0 [0, 4.2]	66	25.8 [15.8, 38.0]	110	4.5 [1.5, 10.3]	8.2 [3.8, 15.0]

* One-sided, 97.5% confidence interval.

## DISCUSSION

The results of TAS conducted in 2013 in The Gambia indicated that there was no LF transmission among 6- and 7-year-old children, and in 2016, The Gambia was removed from the WHO’s official list of LF-endemic countries.^4^ Although the absence of antigenemia among children in The Gambia was likely an indicator of interrupted transmission, there was no information collected from older age groups. Our study aimed to assess antifilarial antibody levels among populations living in historically highly LF-endemic areas in The Gambia and to evaluate the use of serologic tools to confirm the absence of LF transmission.

Overall, no antigenemia was detected, and the prevalence of antibodies to Wb123 was low in the 15 villages included in the survey conducted in 2015. Although there was clear evidence of LF transmission in the 1970s,^18^ results from the current survey suggest that little to no transmission of LF was occurring in these areas, consistent with the results of TAS implemented in The Gambia in 2013. The dramatic decline in LF prevalence over a 50-year period has been observed with increasingly sensitive diagnostic markers and strongly suggests that sustainable LF transmission likely ceased in The Gambia during this period. The decrease in LF prevalence has been mainly attributed to a reduction in mosquito density because of changes in climate, improved standard of living, and the use of bednets for protection from mosquito bites because no LF-specific interventions have taken place.^18^ The rapid scale-up of insecticide-treated nets used for malaria control since 2000 has likely further contributed to the decline.^22,23^ Antibody responses are generally considered an early and sensitive indicator of transmission, and although there is incomplete information about the duration of antibody responses, they do seem to fall as transmission declines. In a study conducted in the Cook Islands, samples collected in the mid-1970s were analyzed for antibodies to Wb123 and compared with results from samples collected from the same island in 1992, 5 years after a single round of MDA against LF. Results indicated a significant decrease in Wb123 antibody positivity, suggesting that LF transmission had significantly decreased.^24^ Recently, in Indonesia, antibody responses were used to successfully distinguish areas where programs had been implemented and successful, suboptimally implemented, and not implemented at all.^25^ Although there is a need to gain more practical experience to operationalize the use of antibody assays, including determining the most appropriate diagnostic platform (e.g., rapid test, ELISA), our results have added information on the utility of serologic tools in an area where MDA was never conducted.

Although seroprevalence was low, positive responses were not completely absent. Antibody responses could represent very focal areas of persistent or recurrent LF transmission, residual seropositivity after interruption of transmission, cross-reactivity, or false-positive results; the detected Wb123 responses may have different implications than the detected responses to Bm14. Approximately, half of the villages had at least one positive Wb123 response. Six of the eight villages with at least one antibody-positive individual were located in the Western Division, where mf rates were in excess of 50% in the 1950s.^17^ However, the absence of positive antigen tests in these villages makes it less likely that these results reflect focal transmission.

If positive serology reflected persistence after the interruption of transmission, an association with age would be expected.^8,26,27^ Antibodies to Bm14 can persist for years, but the expectation is that seroreversion will occur at some point.^28,29^ However, currently, there is insufficient data available on the rates of antibody decay to accurately predict when filarial infection cleared. An association between seropositivity and age was seen for the responses to Bm14, but not to Wb123; in Tambasansang, Bm14-positive individuals were all older than 50 years and could have been exposed to infected mosquitoes before transmission had ceased in the country.

The recombinant Bm14 antigen has also been reported as a highly sensitive marker for the assessment of filarial antibodies, but it is also known to cross-react with closely related filarial parasites.^13,14^ Wb123, used on various diagnostic platforms including ELISAs, is reported to have high sensitivity and specificity for distinguishing *W. bancrofti* infection from closely related filarial infections.^15^ However, a possible explanation for the detected positive Wb123 responses is lower than expected Wb123 specificity. It is possible that the cutoff values for the ELISAs were inaccurate. The ability to define robust cutoffs for serological assays can be challenging and is often limited by the availability of well-characterized panels of samples to determine appropriate cutoffs.

As the GPELF continues to make progress, it is critical to identify strategies for reaching stated goals. Our results strongly suggest that LF transmission has likely ceased in The Gambia and that no programmatic intervention is required. Although there is a clear need to better understand the limitations of current antibody tests, to develop appropriate sampling strategies, and to determine optimal age groups to define antibody thresholds to provide robust evidence of the absence of transmission, our results also support the use of antibody tools to determine the status of LF transmission and suggest that serologic tools can have a role in guiding programmatic decision making.

## References

[1] Taylor MJ Hoerauf A Bockarie M, 2010 Lymphatic filariasis and onchocerciasis. Lancet 376: 1175–1185. 20739055 10.1016/S0140-6736(10)60586-7

[2] WHO, 1997 Elimination of lymphatic filariasis as a public health problem. In: *Fiftieth World Health Assembly* (50.29), Vol. III, 3rd edition. Geneva, Switzerland: World Health Organization.

[3] Ottesen EA, 2006 Lymphatic filariasis: treatment, control and elimination. Adv Parasitol 61: 395–441. 16735170 10.1016/S0065-308X(05)61010-X

[4] WHO, 2016 Global programme to eliminate lymphatic filariasis: progress report, 2015. Wkly Epidemiol Rec 91: 441–455. 27758091

[5] WHO, 2011 *Monitoring and Epidemiological Assessment of Mass Drug Administration in the Global Programme to Eliminate Lymphatic Filariasis: A Manual for National Elimination Programmes.* Geneva, Switzerland: World Health Organization.

[6] Ottesen EA Duke BO Karam M Behbehani K, 1997 Strategies and tools for the control/elimination of lymphatic filariasis. Bull World Health Organ 75: 491–503. 9509621 PMC2487030

[7] Weil GJ Ramzy RM, 2007 Diagnostic tools for filariasis elimination programs. Trends Parasitol 23: 78–82. 17174604 10.1016/j.pt.2006.12.001

[8] Gass K 2012 A multicenter evaluation of diagnostic tools to define endpoints for programs to eliminate *Bancroftian filariasis*. PLoS Negl Trop Dis 6: e1479. 22272369 10.1371/journal.pntd.0001479 PMC3260316

[9] Weil GJ Lammie PJ Weiss N, 1997 The ICT filariasis test: a rapid-format antigen test for diagnosis of *Bancroftian filariasis*. Parasitol Today 13: 401–404. 15275155 10.1016/s0169-4758(97)01130-7

[10] Weil GJ 2013 Laboratory and field evaluation of a new rapid test for detecting *Wuchereria bancrofti* antigen in human blood. Am J Trop Med Hyg 89: 11–15. 23690552 10.4269/ajtmh.13-0089 PMC3748464

[11] Hamlin KL Moss DM Priest JW Roberts J Kubofcik J Gass K Streit TG Nutman TB Eberhard ML Lammie PJ, 2012 Longitudinal monitoring of the development of antifilarial antibodies and acquisition of *Wuchereria bancrofti* in a highly endemic area of Haiti. PLoS Negl Trop Dis 6: e1941. 23236534 10.1371/journal.pntd.0001941 PMC3516578

[12] Muck AE Pires ML Lammie PJ, 2003 Influence of infection with non-filarial helminths on the specificity of serological assays for antifilarial immunoglobulin G4. Trans R Soc Trop Med Hyg 97: 88–90. 12886811 10.1016/s0035-9203(03)90033-2

[13] Lammie PJ Weil G Noordin R Kaliraj P Steel C Goodman D Lakshmikanthan VB Ottesen E, 2004 Recombinant antigen-based antibody assays for the diagnosis and surveillance of lymphatic filariasis–a multicenter trial. Filaria J 3: 9. 15347425 10.1186/1475-2883-3-9 PMC519021

[14] Weil GJ Curtis KC Fischer PU Won KY Lammie PJ Joseph H Melrose WD Brattig NW, 2011 A multicenter evaluation of a new antibody test kit for lymphatic filariasis employing recombinant *Brugia malayi* antigen Bm-14. Acta Trop 120 (Suppl 1): S19–S22. 20430004 10.1016/j.actatropica.2010.04.010 PMC2935504

[15] Kubofcik J Fink DL Nutman TB, 2012 Identification of Wb123 as an early and specific marker of *Wuchereria bancrofti* infection. PLoS Negl Trop Dis 6: e1930. 23236529 10.1371/journal.pntd.0001930 PMC3516582

[16] Steel C Golden A Kubofcik J LaRue N de Los Santos T Domingo GJ Nutman TB, 2013 Rapid *Wuchereria bancrofti*-specific antigen Wb123-based IgG4 immunoassays as tools for surveillance following mass drug administration programs on lymphatic filariasis. Clin Vaccine Immunol 20: 1155–1161. 23740923 10.1128/CVI.00252-13 PMC3754496

[17] McFadzean J, 1954 Filariasis in Gambia and Casamance, West Africa. Trans R Soc Trop Med Hyg 48: 267–273. 13169245 10.1016/0035-9203(54)90075-9

[18] Knight R, 1980 Current status of filarial infections in The Gambia. Ann Trop Med Parasitol 74: 63–68. 6990885 10.1080/00034983.1980.11687311

[19] Rebollo MP Sambou SM Thomas B Biritwum NK Jaye MC Kelly-Hope L Escalada AG Molyneux DH Bockarie MJ, 2015 Elimination of lymphatic filariasis in The Gambia. PLoS Negl Trop Dis 9: e0003642. 25785587 10.1371/journal.pntd.0003642 PMC4364952

[20] Pavluck A Chu B Mann Flueckiger R Ottesen E, 2014 Electronic data capture tools for global health programs: evolution of LINKS, an Android-, web-based system. PLoS Negl Trop Dis 8: e2654. 24722343 10.1371/journal.pntd.0002654 PMC3983089

[21] Chandrashekar R Curtis KC Ramzy RM Liftis F Li BW Weil GJ, 1994 Molecular cloning of *Brugia malayi* antigens for diagnosis of lymphatic filariasis. Mol Biochem Parasitol 64: 261–271. 7935604 10.1016/0166-6851(94)00035-2

[22] Ceesay SJ 2008 Changes in malaria indices between 1999 and 2007 in The Gambia: a retrospective analysis. Lancet 372: 1545–1554. 18984187 10.1016/S0140-6736(08)61654-2 PMC2607025

[23] Noor AM Mutheu JJ Tatem AJ Hay SI Snow RW, 2009 Insecticide-treated net coverage in Africa: mapping progress in 2000–07. Lancet 373: 58–67. 19019422 10.1016/S0140-6736(08)61596-2 PMC2652031

[24] Steel C Kubofcik J Ottesen EA Nutman TB, 2012 Antibody to the filarial antigen Wb123 reflects reduced transmission and decreased exposure in children born following single mass drug administration (MDA). PLoS Negl Trop Dis 6: e1940. 23236533 10.1371/journal.pntd.0001940 PMC3516579

[25] Dewi RM Tuti S Ganefa S Anwar C Larasati R Ariyanti E Herjati H Brady M, 2015 Brugia Rapid antibody responses in communities of Indonesia in relation to the results of ‘transmission assessment surveys’ (TAS) for the lymphatic filariasis elimination program. Parasit Vectors 8: 499. 26427536 10.1186/s13071-015-1093-x PMC4589901

[26] Mladonicky JM King JD Liang JL Chambers E Pa’au M Schmaedick MA Burkot TR Bradley M Lammie PJ, 2009 Assessing transmission of lymphatic filariasis using parasitologic, serologic, and entomologic tools after mass drug administration in American Samoa. Am J Trop Med Hyg 80: 769–773. 19407122

[27] Shawa ST Mwase ET Pedersen EM Simonsen PE, 2013 Lymphatic filariasis in Luangwa district, south-east Zambia. Parasit Vectors 6: 299. 24499525 10.1186/1756-3305-6-299 PMC3853755

[28] Ramzy RM El Setouhy M Helmy H Ahmed ES Abd Elaziz KM Farid HA Shannon WD Weil GJ, 2006 Effect of yearly mass drug administration with diethylcarbamazine and albendazole on *Bancroftian filariasis* in Egypt: a comprehensive assessment. Lancet 367: 992–999. 16564361 10.1016/S0140-6736(06)68426-2

[29] Moss DM Priest JW Boyd A Weinkopff T Kucerova Z Beach MJ Lammie PJ, 2011 Multiplex bead assay for serum samples from children in Haiti enrolled in a drug study for the treatment of lymphatic filariasis. Am J Trop Med Hyg 85: 229–237. 21813840 10.4269/ajtmh.2011.11-0029 PMC3144818

